# Comparison of external stents and DJ stents techniques for pediatric pyeloplasty: A systematic review and meta-analysis

**DOI:** 10.3389/fped.2022.933845

**Published:** 2022-08-25

**Authors:** Chunyang Meng, Lijian Gan, Kangsen Li, Lei Peng, Jinze Li, Junbao Yang, Yunxiang Li

**Affiliations:** ^1^Department of Urology, The Affiliated Nanchong Central Hospital of North Sichuan Medical College (University), Nanchong, China; ^2^Department of Urology, The Second Hospital of Lanzhou University Medical School, Lanzhou, China; ^3^Department of Urology, Institute of Urology, West China Hospital, Sichuan University, Chengdu, China; ^4^Department of Medical Genetics and Cell Biology, North Sichuan Medical College (University), Nanchong, China

**Keywords:** UPJO, external stent, Double J, pediatric pyeloplasty, meta-analysis

## Abstract

**Objective:**

To evaluate and compare the efficacy and safety between an external stent and a Double J stent for pediatric Pyeloplasty.

**Methods:**

Through a systematical search of multiple scientific databases in July 2022, we performed a systematic review and meta-analysis of the primary outcomes of interest according to the PRISMA (Preferred Reporting Items for Systematic Reviews and Meta-Analyses), whose protocol was registered with PROSPERO(CRD42021274087).

**Results:**

Eleven studies involving 1,758 patients were included. No significant differences were observed in operative time (MD: 2.26; 95% CI −9.62 to 14.14; *P* = 0.79), operative success rate (OR: 1.10; 95% CI 0.57 to 2.10; *P* = 0.780), length of hospital stay (MD: 0.65; 95% CI −0.04 to 1.34; *P* = 0.063), or complications (OR: 0.87; 95%CI 0.48 to 1.56; *P* = 0.630) between external stents and DJ stents in pediatric pyeloplasty. According to the subgroup analysis, we found the external stent group had a shorter operative time than the DJ stent group in terms of robot-assisted laparoscopic pyeloplasty (MD: −17.13; 95% CI −32.8 to −1.45; *P* = 0.032).

**Conclusions:**

There were no significant differences in operative time, operative success rate, length of hospital stay, or complications between external stents and DJ stents in pediatric pyeloplasty. The external stented procedure seemed to have less operative time when using robot-assisted laparoscopic pyeloplasty. However, due to the limitations of our analysis, more studies are still required to support our conclusion.

**Systematic review registration:**

This systematic review has been registered on PROSPERO, the registration ID is CRD42021274087.

## Introduction

The most common congenital abnormality of the upper urinary tract is Uretero-Pelvic Junction Obstruction (UPJO) and the incidence in newborns is 1 in 1,500. Approximately 20–50% of children with hydronephrosis will eventually undergo surgical intervention (1). Since it was first described, Anderson-Hynes dismembered pyeloplasty has been considered the standard surgical treatment for UPJO (2). Not only for open pyeloplasty but also minimally pyeloplasty, encouraging outcomes have been demonstrated (3–5). The management of anastomosis drainage after pyeloplasty includes no stent, internal Double J(DJ) stent, and external stent. Although researchers have described outstanding outcomes of unstented pyeloplasty (6, 7), a large number of surgeons still prefer to stent the newly formed anastomosis.

Several methods of postoperative renal drainage have been described, and internal DJ stents or external stents are used most often. DJ stents are usually placed in an antegrade manner, and the size is chosen by surgeon preference. The most commonly used size is 4.7 French in pediatric pyeloplasty. Kidney-ureter-bladder X-ray was performed the next day to confirm the position (8). DJ stents are not easy to insert, and their use may be accompanied by the risk of stent migration, urinary tract infection, and second anesthesia for removal (9). The external stent is passed from the ureteropelvic junction to the skin through the kidney parenchyma, and the lower end of the stent reaches the mid or lower ureter. It is fixed with sutures on the renal capsule or skin to reduce the risk of displacement (10). An external stent has the advantage that it can be removed in the outpatient department without anesthesia. However, the risk of bleeding and stent leakage cannot be ignored when using an external stent (4, 11).

Currently, the type of stents to place in clinical practice mainly depends on the surgeon's preference and experience. To fill this gap, we performed a meta-analysis to assess and compare the efficacy and safety between external stents and DJ stents.

## Methods

### Literature search and eligibility criteria

Based on the guidelines of Preferred Reporting Items for Systematic Reviews (12), a systematic search was performed to identify studies in PubMed, Embase, Scopus database, and Cochrane Library. The latest search data was July, 2022.Search terms included “pediatric,” “double J,” “tube,” “DJ,” “stent^*^,” “external^*^,” “UPJO,” “Anderson-Hynes,” “pyeloplasty,” etc. We also broadened the search scope by manual retrieval, and the search was not restricted by language.

The studies were included in our meta-analysis if they met the following criteria: (1) patients were diagnosed with UPJO and pyeloplasty was performed, (2) the comparison between external stents and DJ stents, (3) the full paper was full of no < 1 outcome parameter. The exclusion criteria were as follows: (1) patients with another disease except for UPJO, (2) previous history of pyeloplasty, (3) reviews, case reports, letters, and irrelevant studies about our topic, (4) available data could not be extracted.

### Data extraction

The extraction process was independently completed by two authors (CY.M and L.P), and the following information was recorded: author, publication year, country, study design, sample size, inventions, mean age, follow-up time, operative time, operative success rate, length of hospital stay, and complications. When continuous variables were described as other forms in the main literature, we calculated the mean and standard deviation (13).

### Study quality assessment

Randomized controlled trials were assessed by the Jadad scale (14) and non-randomized controlled trials were evaluated by the NOS scoring rules (15). The Jadad score varied up to seven points, and more than four points were graded as high quality. The NOS scale ranged from zero to nine stars, and scores of more than six stars were considered high quality.

### Risk of bias assessments

The ROBINS-I tool was used to evaluate the risk of bias for non-randomized studies. The ROBINS-I tool included seven domains: confounding bias, selection bias, bias in the measurement classification of interventions, bias due to deviations from intended interventions, bias due to missing data, bias in the measurement of outcomes, and bias in the selection of the reported result (16). Moreover, we used the ROB2 tool to evaluate the risk of bias for randomized controlled trials. ROB2 covered the randomization process, deviations from intended interventions, missing outcome data, measurement of the outcome, and selection of the reported result (17).

### Data analysis

We accomplished data analysis by using STATA version 16.0. the mean difference (MD) and odds ratio (OR) were considered continuous and dichotomous outcomes, respectively. In addition, we calculated a 95% confidence interval (CI) and used I-square tests to verify the heterogeneity among the included studies. Statistical significance was defined as *P* < 0.05. Subgroup analysis was performed according to different surgical methods, such as open pyeloplasty, laparoscopic pyeloplasty, and robot-assisted laparoscopic pyeloplasty.

## Results

### Description of studies

**Figure 1:**
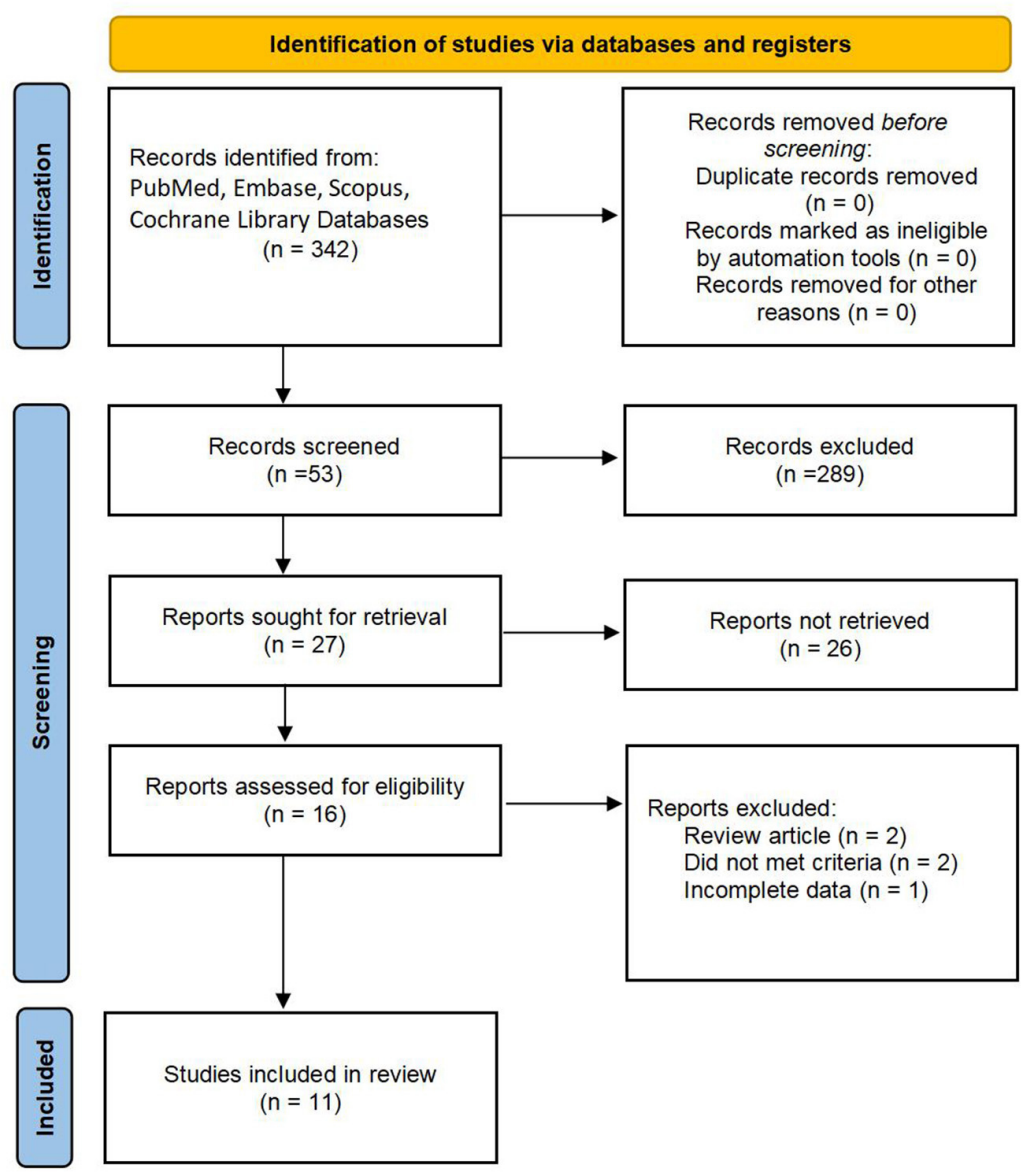
Flow diagram of the studies selection process.

A total of 342 studies were identified, and eleven studies were eventually included in our meta-analysis (3, 4, 8–11, 18–22). The filtering process is shown in Figure 1. The baseline characteristics of the included studies are given in Table 1 eleven studies, including 1,758 patients, were published between 2008 and 2021. Moreover, the sample size ranged from 22 to 650. Among them, ten types of research were retrospective designs, and one was a randomized controlled trial.

**Table 1:** Baseline characteristic of included studies.

**References**	**Country**	**Study design**	**Sample**	**Inventions**	**Groups sample**	**Mean age**	**Male/Female**	**Follow-up**	**Quality score**	**Outcomes**
Braga et al. (18)	Canada	Retrospective	470	External stented	228	18 y	150/78	39 mos	7^e^	b, d
				DJ stented	242	19 y	137/105	41 mos		
Helmy et al. (9)	France	Retrospective	22	External stented	11	31 mos	NA	34 mos	7^e^	a, b, c, d
				DJ stented	11	37 mos	NA	35 mos		
Zoeller et al. (21)	Germany	Retrospective	86	External stented	38	5.6 y	26/12	12 mos	7^e^	a, c, d
				DJ stented	48	5.6 y	36/12			
Kocvara et al. (19)	Czech	Retrospective	36	External stented	15	34 mos	10/5	36.2 mos	8^e^	a, d
				DJ stented	21	46 mos	11/4			
Lee et al. (4)	Canada	Retrospective	62	External stented	24	40 mos	16/8	23.8 mos	7^e^	a, b, c, d
				DJ stented	38	80 mos	29/9	21.1 mos		
Garg et al. (11)	India	RCT	40	External stented	20	3.76 y	NA	≥3 mos	4^f^	b, c, d
				DJ stented	20	2.7 y	NA			
Chu et al. (3)	USA	Retrospective	61	External stented	17	8 y	NA	12.3 mos	8^e^	a, b, c, d
				DJ stented	44	7.9 y	NA	12.1 mos		
Lombardo et al. (20)	USA	Retrospective	103	External stented	33	3.91 y	29/4	21.2 mos	7^e^	a, b, c, d
				DJ stented	70	7.61 y	50/20	23.4 mos		
Paraboschi et al. (8)	UK, Italy	Retrospective	53	External stented	27	58.8 mos	13/14	26.3 mos	8^e^	a, b, c, d
				DJ stented	26	107.2 mos	13/13	31.4 mos		
Sarhan et al. (10)	Egypt	Retrospective	175	External stented	65	40 mos	42/23	48 mos	8^e^	a, c, d
				DJ stented	110	37 mos	78/32			
Kong et al. (22)	China	Retrospective	650	External stented	107	48 mos	79/28	≥12 mos	8^e^	a, d
				DJ stented	543	57 mos	445/98			

a, operative time; b, operative success rate; c, length of hospital stay; d, complications; e, using NOS scoring rules; f, using Jadad scale; RCT, randomized controlled trial.

### Quality assessment

The quality of the randomized controlled trials was relatively high (Jadad scale: 4 points). For non-randomized controlled trials, five studies scored seven stars and four scored eight stars. The ultimate quality assessment list is shown in Table 1.

### Risk of bias

The major weakness of randomized controlled trials was in the domains of randomization process and deviations from intended interventions, as shown in Supplementary Figure 1. For non-randomized controlled trials, the final results showed that eight studies were at moderate risk of bias and one was low. The outcome was provided in the Supplementary Table 1.

### Operative time

**Figure 2:**
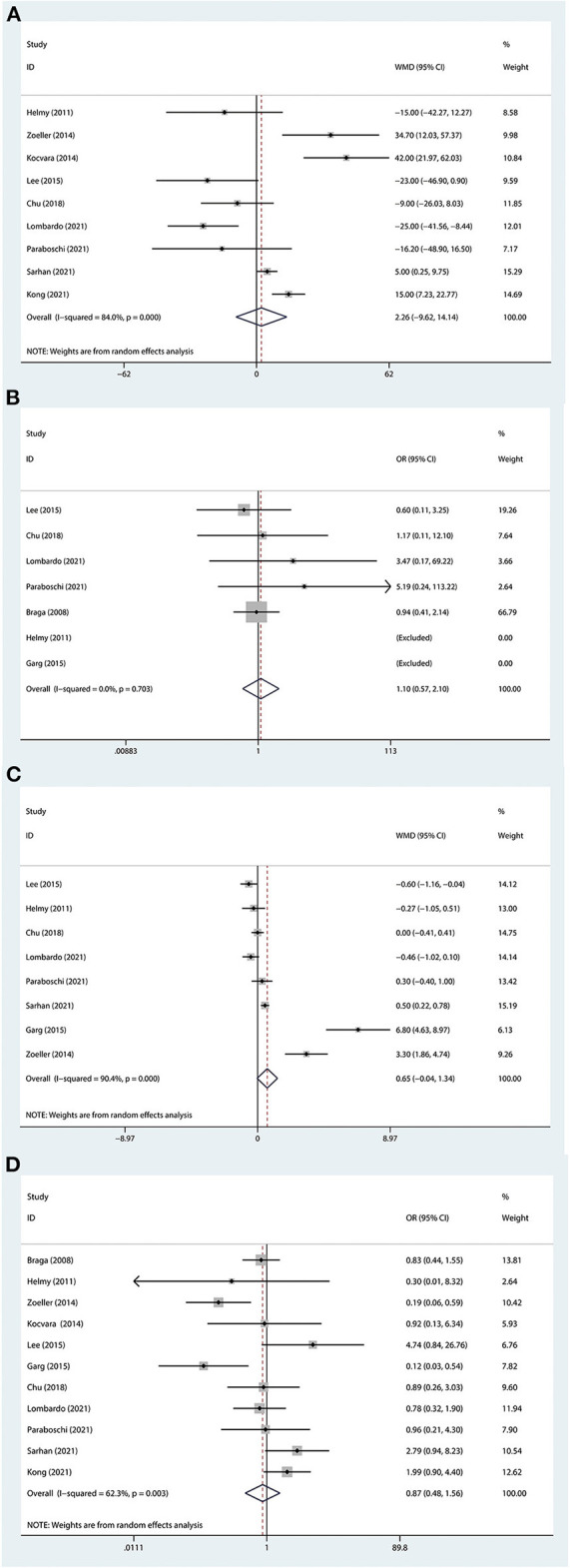
Forest plot of perioperative outcomes. **(A)** Operative time. **(B)** Operative success rate. **(C)** Length of hospital stay. **(D)** Complication. The horizontal lines represent 95% CI. The intersection of diamond and vertical line means that the difference is not statistically significant.

Nine studies with 1,287 patients were related to operative time (3, 4, 8–10, 19–22). Due to the high heterogeneity (I^2^>50%), we used the random-effects model. The pooled meta-analysis results showed that no significant difference was found between the external stent group and the DJ group (MD: 2.26; 95% CI −9.62 to 14.14; *P* = 0.79; Figure 2A).

### Operative success rate

The results of the surgery were stated in 7 studies (3, 4, 8, 9, 11, 18, 20), and all included groups reached a high success rate. Based on heterogeneous results (I^2^ = 0%), the fixed effects model was used. The final results indicated that no significant difference was found between the two groups (OR: 1.10; 95% CI 0.57 to 2.10; *P* = 0.780; Figure 2B).

### Length of hospital stay

The length of hospital stay was observed in 8 studies (3, 4, 8–11, 20, 21), including 602 patients. Given the heterogeneity test outcome (I^2^>50%), we used the random-effects model. The ultimate result showed that there was no difference between the external stent group and the DJ group (MD: 0.65; 95% CI −0.04 to 1.34; *P* = 0.063; Figure 2C).

### Complications

**Figure 3:**
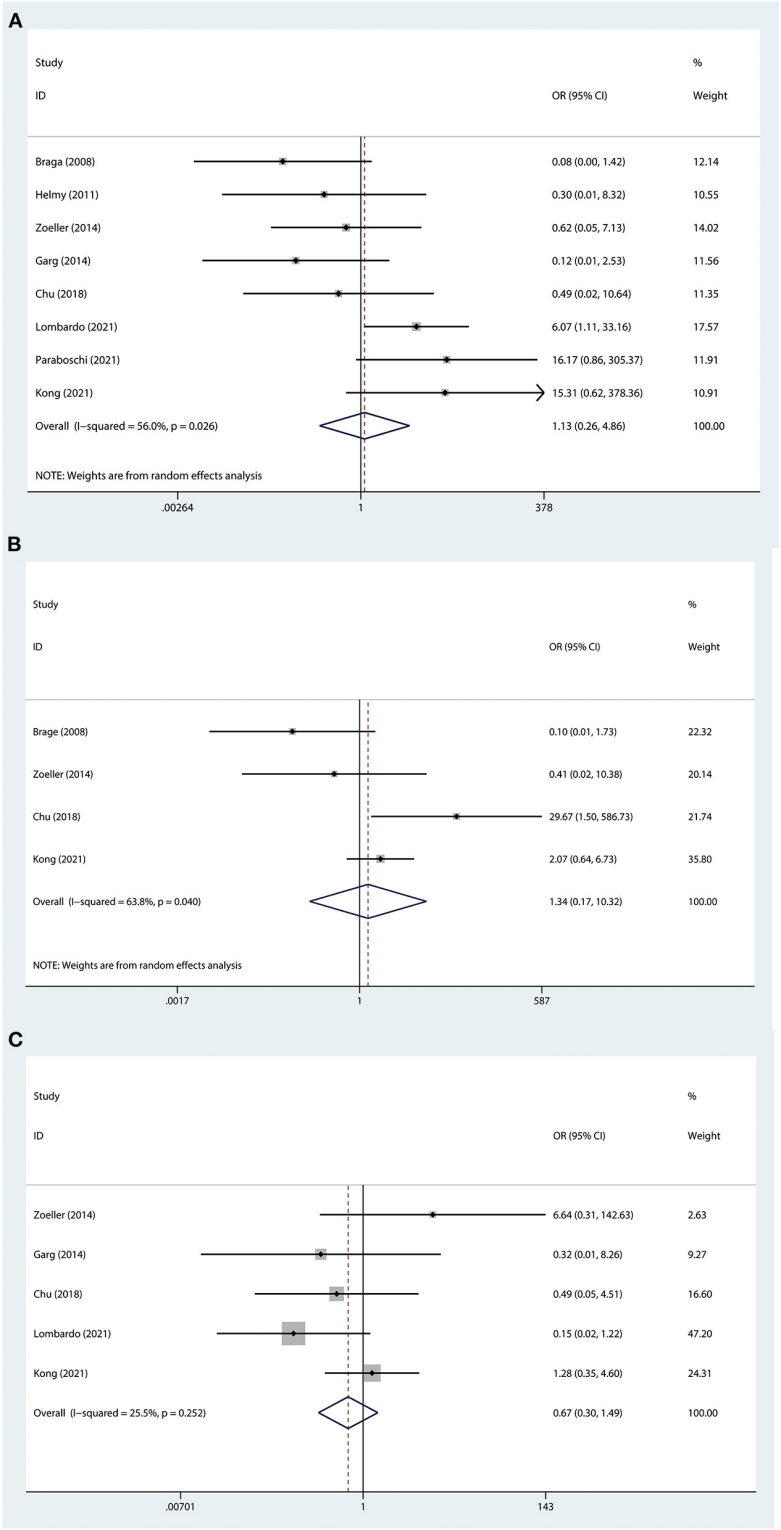
Forest plot of major complication. **(A)** stent dislodgement. **(B)** stent leakage. **(C)** urinary tract infection. The horizontal lines represent 95% CI. The intersection of diamond and vertical line means that the difference is not statistically significant.

Eleven studies had been linked to complications (3, 4, 8–11, 18–22), concerning 1,758 patients. Because of the heterogeneous results (I^2^ = 62.3%), a random effect model was applied. The overall meta-analysis showed that there was no difference between the two groups in terms of complications (OR: 0.87; 95%CI 0.48 to 1.56; *P* = 0.630; Figure 2D). Moreover, some major complications, which included stent dislodgement, stent leakage, and urinary tract infection, were analyzed and there was no significant difference (Figure 3).

### Subgroup analysis

Subgroup analysis was performed according to sample size, publication year, and surgical methods, Owing to the lack of sufficient data, the subgroup analysis could not be performed on major complications. The last outcome of the subgroup analysis recommended that the external stent group had a shorter operative time than the DJ stent group in terms of robot-assisted laparoscopic pyeloplasty (MD: −17.13; 95% CI −32.8 to −1.45; *P* = 0.032; Table 2).

**Table 2:** Subgroup analysis of included studies.

**Subgroup**	**Operative time**			**Operative success rate**			**Length of hospital stay**			**Postoperative complications**		
	**95% CI**	**I^2^ (%)**	***P***	**95% CI**	**I^2^ (%)**	***P***	**95% CI**	**I^2^ (%)**	***P***	**95% CI**	**I^2^ (%)**	***P***
Sample size												
>80	6.49 (−8.07, 21.04)	87.9	0.382	1.07 (0.49, 232)	0	0.863	0.87 (−0.37, 2.10)	92	0.170	0.95(0.44, 2.05)	74.2	0.891
<80	−3.36 (−28.59, 21.86)	83.1	0.794	1.16 (0.36, 3.78)	0	0.808	0.66 (−0.36, 1.69)	90.8	0.206	0.76 (0.27, 2.09)	52.7	0.592
Publication year												
≥2015	−5.46 (−17.57, 6.65)	82.3	0.377	1.41 (0.48, 4.16)	0	0.530	0.45 (−0.28, 1.18)	90.7	0.225	1.14 (0.54, 2.38)	63.7	0.731
<2015	21.66 (−10.68, 54.01)	82.9	0.189	0.94 (0.41, 2.14)	0	0.881	1.46 (−2.04, 4.96)	94.5	0.413	0.50 (0.20, 1.22)	44.2	0.128
Surgical method												
OP	−6.45 (−33.43, 20.53)	80.3	0.639	0.86 (0.41, 1.81)	0	0.696	1.63 (−0.14, 3.41)	95.7	0.071	1.05 (0.30, 3.76)	79.4	0.935
LP	14.64 (−4.09, 33.38)	77.5	0.125	5.19 (0.24, 113.22)	0	0.295	0.98 (−0.62, 2.57)	89.1	0.232	0.71 (0.23, 2.17)	66.3	0.549
RALP	−17.13 (−32.8, −1.45)	42.6	0.032	1.92 (0.32, 11.64)	0	0.480	−0.19 (−0.63, 0.25)	40.6	0.401	0.82 (0.40, 1.68)	0	0.586

OP, open pyeloplasty; LP, laparoscopic pyeloplasty; RALP, robot assisted laparoscopic pyeloplasty.

### Sensitivity analysis

**Figure 4:**
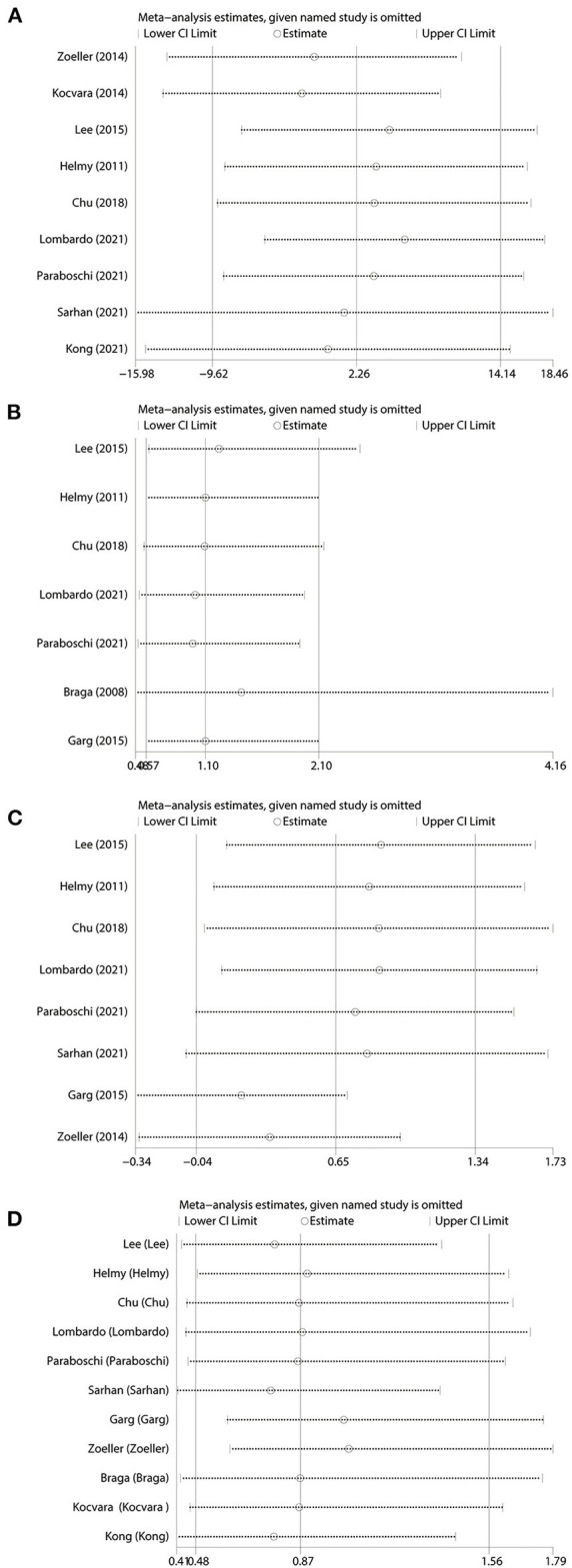
Sensitivity analysis. **(A)** Operative time. **(B)** Operative success rate. **(C)** Length of hospital stay. **(D)** Complication. Studies are removed one by one to recalculate the combined effect size, and compare the difference with the initial effect size.

Seeing the outcomes of heterogeneity, we performed a sensitivity analysis to improve the reliability of the results. Studies were removed one by one to recalculate the combined mean difference, and the outcomes of our meta-analysis were stable except for the length of hospital stay (Figure 4).

## Discussion

Even if the safety and efficacy of unstented pyeloplasty were reported, the majority of surgeons prefer to place a trans anastomotic stent to drain urine. This could help to release the stress on the newly formed anastomosis (23). The DJ stent and external stent were the main management of anastomosis drainage after pyeloplasty, and each measure had its advantages and disadvantages (24, 25). The major shortcoming of the DJ stent was that patients, especially infants, needed to undergo second general anesthesia when withdrawing it. Nevertheless, doctors can remove external stents through the renal parenchyma or the renal pelvis even without general anesthesia.

Eleven comparative studies, including 1,758 patients, were eventually included in our meta-analysis. Based on the outcomes, we observed that in the terms of operative time, operative success rate, length of hospital stay, and postoperative complications, no significant differences were found. According to the outcome of the subgroup analysis, the external stent group had a shorter operative time than the DJ stent group in terms of robot-assisted pyeloplasty.

In terms of operative time, our conclusion was consistent with most of the previous outcomes, which meant there was no significant difference between the two groups. As a result of the three operative approaches, subgroup analysis was performed by dividing the patients into three groups. The results of the subgroup analysis suggested that only in robot-assisted laparoscopic pyeloplasty did patients in the external stent group have a shorter operative time than those in the DJ stent group. This might mean that, to some extent, the external stent was not second to the DJ stent, especially in robot-assisted laparoscopic pyeloplasty.

Operative success was defined as the improvement of clinical symptoms and radiological evidence of improvement of hydronephrosis. The operative success rate in both two groups was very high, with a range of 87.5–100% in the external stent group and the range of 92.1–100% in the DJ group. No differences were considered significant in our meta-analysis. A recent study of 85 children aged 0–16 years, compared the changes in differential renal function after pyeloplasty in children and showed that most children had preserved or improved renal function after surgery (26). Combining the above evidence, showed similar effectiveness between the two drainage techniques despite the use of different surgical techniques.

For the length of hospital stay, the final result demonstrated that there was no difference between the external stent group and the DJ group. Nevertheless, in some studies, patients in the external stent group stayed longer in the hospital (10, 11, 21). As far as we know, the use of a DJ stent was associated with a lower risk of urinary leakage, thereby enabling patients to recover rapidly. In addition, if successful clamping or poor drainage is lacking, hospitalization for patients with external stents might be longer (11). In Professor Zoeller's study, he pointed out that doctors' decisions on discharge time were affected by parents' fear or inability to manage external drainage (21). This also might prolong hospitalization.

Concerning the result of overall complications, we did not find significant differences between the external stent group and the DJ stent group, which was consistent with most previous studies (3, 4, 18). Some studies have suggested that several postoperative problems can be avoided by choosing a suitable stent length and some tools were offered for choice (27, 28). To avoid the excess length of the stent in the bladder, Professor Garg proposed the formula: Length of DJ stent (cm) = length of the retained ureter (cm)−2. Even so, 9 of 20 patients in the DJ group experienced an increase in the frequency of urination. In contrast, only one person had frequent urination in the control group (11). In Professor Zoeller's study, the main technical problem was the inability to place a DJ stent in infants (21).

Stent dislodgement, stent leakage, and urinary tract infection were the major complications. In a study involving 55 patients, there were 5(9.1%) cases of DJ stent dislodgement, including 3 DJ stents migrating into the bladder and 2 into the upper urinary tract (29). In the study reported by Chu et al., the incidence of DJ stent displacement (4.5%) was higher than that of the external stent (0%) (3). Furthermore, DJ stents in the ureter could cause artificial vesicoureteral reflux and have been associated with a higher incidence of urinary tract infection (3, 19). In addition, DJ stents were forgotten also to be a matter worth the attention of clinicians. The use of external stents avoids bladder-related symptoms, but it may be associated with a higher incidence of stent leakage, bleeding, and skin infection (8). Individualized customized stents may help to abate these issues.

One of the advantages that could not be ignored of the external stent was that it avoided the risk of secondary anesthesia. As far as we know, for most infants or children, additional general anesthesia is inevitable when removing DJ tubes (10). Considering the costs related to stent removal, the average cost of the external stent group was £686.7, which was lower than that of the DJ tube group of £1425.6 (8). In another author's findings, regarding open pyeloplasty, the application of an external stent was associated with a Canadian $565 cost decrease per patient (18).

However, there is not yet a standard type for external stents in pediatric pyeloplasty. Most of the available studies use commercial stents, ie Urosoft^®^ multipurpose stent and Salle intraoperative pyeloplasty stent (Cook^®^ Medical), which were accompanied by some degree of modification (8, 10, 20). The external stents are usually modified by removing the distal bladder curl and stents are terminated in the mid or lower ureter, thus effectively avoiding bladder irritation symptoms (3, 20). Professor Zoller et al. have designed a newly constructed spear for transrenal externalized catheter insertion with a blunt tip and a tapered end, which has the advantages of easy positioning and more minor bleeding (21). The fixation of the external stent is important. Almost all studies secure the catheter to the renal capsule so that the risk of stent displacement and exteriorization of the holes is minimized (9).

Although we performed a comprehensive analysis of the efficacy and safety of external stents and DJ stents, several limitations remained. First, the included studies were almost all retrospective studies, and only one RCT was involved. Due to a lack of evidence, more high-level studies are required to support this hypothesis. Second, the external stents of the included studies had numerous different types, which could be associated with heterogeneity. Third, because of the different definitions of renal function improvement and the small numbers reported on different types of complications, we failed to analyze it.

## Conclusion

In conclusion, there were no significant differences in operative time, operative success rate, length of hospital stay, or complications between external stents and DJ stents in pediatric pyeloplasty. The external stented procedure seemed to have less operative time when using robot-assisted laparoscopic pyeloplasty. However, due to the limitations of our analysis, more studies are still required to support our conclusion.

## Data availability statement

The original contributions presented in the study are included in the article/Supplementary material, further inquiries can be directed to the corresponding author/s.

## Author contributions

Conceived and designed the experiments: YL and JY. Analyzed the data: CM, LP, and KL. Contributed reagents, materials, and analysis: LG, KL, and JL. Wrote the manuscript: CM, LP, and LG. All authors have read and approved the final manuscript.

## Funding

This study was supported by Sichuan Science and Technology Program under Grant number 2020YFS0320.

## Conflict of interest

The authors declare that the research was conducted in the absence of any commercial or financial relationships that could be construed as a potential conflict of interest.

## Publisher's note

All claims expressed in this article are solely those of the authors and do not necessarily represent those of their affiliated organizations, or those of the publisher, the editors and the reviewers. Any product that may be evaluated in this article, or claim that may be made by its manufacturer, is not guaranteed or endorsed by the publisher.
