# Supplementary figures and images for: Comparison of external stents and DJ stents techniques for pediatric pyeloplasty: A systematic review and meta-analysis
Source: Front Pediatr. 2022 Aug 25;10:933845. doi: 10.3389/fped.2022.933845 (PMC9452663; doi:10.3389/fped.2022.933845)

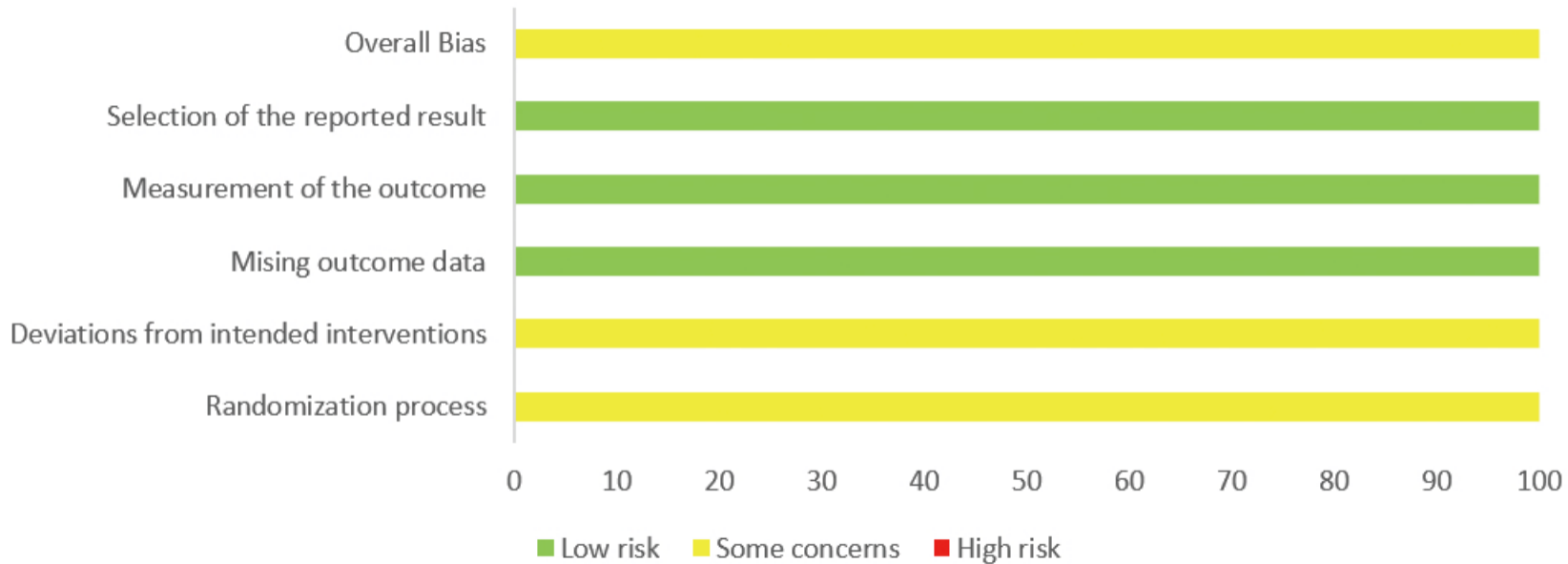

Supplement: Supplementary file 1 [file Image_1.PDF]
