# Supplementary material for: Comparison of external stents and DJ stents techniques for pediatric pyeloplasty: A systematic review and meta-analysis
Source: Front Pediatr. 2022 Aug 25;10:933845. doi: 10.3389/fped.2022.933845 (PMC9452663; doi:10.3389/fped.2022.933845)
Supplement: Supplementary file 2 [file Table_1.DOCX]

Supplementary Table. Risk of Bias for non-randomized studies with ROBINS-I.

| Authors, year | Bias due to  confounding | Selection of  participants | Classification  of interventions | Deviations  from intended  interventions | Bias due to  missing data | Bias in  measurement  of outcomes | Selection of  the reported  result | Overall bias |
| --- | --- | --- | --- | --- | --- | --- | --- | --- |
| Braga et al.2008 | moderate | low | moderate | low | low | low | low | moderate |
| Helmy et al.2011 | low | low | low | low | moderate | low | low | moderate |
| Zoeller et al.2014 | low | low | low | low | moderate | low | low | moderate |
| Kocvara et al.2014 | moderate | low | low | low | low | low | low | moderate |
| Lee et al.2015 | low | moderate | moderate | low | low | low | low | moderate |
| Chu et al.2018 | low | low | low | low | low | low | moderate | moderate |
| Lombardo et al.2021 | low | moderate | low | low | low | low | low | moderate |
| Paraboschi et al.2021 | low | moderate | low | low | low | low | low | moderate |
| Sarhan et al.2021 | low | low | low | low | low | low | low | low |
| Kong et al.2021 | low | moderate | low | low | low | low | low | moderate |
